# Array Comparative Genomic hybridization of breast cancer in the kingdom of Saudi Arabia

**DOI:** 10.1186/1471-2164-15-S2-P66

**Published:** 2014-04-02

**Authors:** Lina Al-Harbi, Ahmed Shokry, Jamal Sabir, Adeel Chaudhary, Ashraf Dallol

**Affiliations:** 1 Center of Excellence in Genomic Medicine Research, King Abdulaziz University, Jeddah 21589, Saudi Arabia; 2 Department of Biological Sciences, Faculty of Science, King Abdulaziz University, P.O. Box 80141 Jeddah 21589, Saudi Arabia; 3 Agricultural Genetic Engineering Research Institute (AGERI), Agriculture Research Center (ARC), Giza, Egypt

## Background

Breast cancer is the second most common cause of cancer death worldwide and is the most common cancer among Saudi females [1]. Breast carcinogenesis is associated by a wide-array of cytogenomic changes involving deletions, amplification or translocations of part or whole chromosome arms. However, there are several changes that could not be detected at the resolution offered by classical cytogenetic techniques. Many of these regions could provide further insight into the disease and may harbor potential prognostic values. The development of Array Comparative Genomic Hybridization (aCGH) allowed the identification of such regions at a genomic scale and currently there is a plethora of scientific publications reporting cytogenomic changes in different cancer types [2]. Unfortunately, there are no studies as yet published specifically looking at breast cancer from Saudi patients.

## Materials and methods

In this study, aCGH was performed on twenty breast cancer tumors and their matching non-tumorous (normal) tissues using the Agilent 2x400K. Several regions were identified to be either amplified or deleted in a tumor-specific manner.

## Results

All samples were obtained from female cancer patients diagnosed with breast cancer and were subjected to breast lumpectomy surgical procedure. The mean age of this group is 51.5 years, range: 35.0-70.1 years. 15 cases were diagnosed with invasive ductal carcinoma (IDC), 2 cases were diagnosed with invasive lobular carcinoma and 2 cases were fibroadenomas. One case was of mucinous carcinoma origin. The main inclusion criteria were the presence of matching non-malignant normal tissue DNA from the same patient to be used as a reference DNA for the aCGH. This is important as it is often difficult to obtain good normal control DNA for breast cancer. The most prevalent chromosome imbalances were chromosome gains of +1q, +8q, +11q, +16q and +20q. Chromosome loss was mostly found in 11p, 13q, 1p, 9q, 16p, 17q, and Xq. In this study, gains were found in 1q25.1, 16q13- 24, 20q11-13, and 8q21-24. Of the DNA copy number losses, 1p36 was most common event, followed by 8p, 16q and 17p.

## Conclusions

This is the first report of high resolution comparative genomic hybridization (aCGH) analysis of breast cancer from Saudi Arabia. We demonstrate our ability to detect known regions of amplification and deletions that occur in a tumor-specific manner. This finding serves as a validation to our approach as we are able to compare our findings to published benchmarks. We further demonstrate the potential importance of aCGH in breast cancer diagnosis as, through the detection of copy number changes, a patient sample can be characterized in terms of the cytogenetic aberrations it harbors. The detection of 1q, 1p, 8q amplification events by aCGH and FISH suggests that a considerably larger study can be established where as a significantly larger cohort with clinicopathological data can be analyzed using only FISH analysis. Prognostic biomarkers can then be identified if correlations could be made with copy number variations in these regions and known clinicopathological parameters, especially recurrence and survival.
